# Cholesterol Drives IFITM3 ^+^ Microglia Activation and Induces STING Mediated Neuroinflammation After Ischemic Stroke

**DOI:** 10.1002/cns.71073

**Published:** 2026-08-11

**Authors:** Yue Cheng, Yuxi Zhou, Yonghui Chen, Tianni Shen, Yan Li, Chen Chen, Qiuyue Fan, Jie Qi, Peiying Li, Yueman Zhang

**Affiliations:** ^1^ Department of Anesthesiology Renji Hospital, Shanghai Jiao Tong University School of Medicine Shanghai China; ^2^ Key Laboratory of Anesthesiology Shanghai Jiao Tong University, Ministry of Education Shanghai China; ^3^ Clinical Research Center Renji Hospital, Shanghai Jiao Tong University School of Medicine Shanghai China

**Keywords:** cholesterol, ischemic stroke, microglia, neuroinflammation, STING

## Abstract

**Aims:**

Cerebral ischemic stroke triggers extensive neuronal membrane breakdown, releasing a massive load of cholesterol that overwhelms resident microglia. Dysregulated microglial cholesterol metabolism has been implicated in post‐stroke neuroinflammation, yet the specific pathogenic microglial subpopulations, their molecular signatures, and the downstream inflammatory cascades remain poorly defined.

**Methods:**

We employed a permanent distal middle cerebral artery occlusion (dMCAO) model combined with single‐cell RNA sequencing (scRNA‐seq) to profile immune cell transcriptomes and identify cholesterol‐associated microglial markers. Cholesterol dynamics, lipid droplet accumulation, and inflammatory marker expression were quantified via immunofluorescence and transmission electron microscopy. Therapeutic interventions included pharmacological cholesterol mobilization with 2‐hydroxypropyl‐β‐cyclodextrin (HβCD), pharmacological STING inhibition with C‐176, and microglia‐targeted STING knockdown using AAV9 vectors. Cerebral injury and neurological function were assessed through infarct volume measurement, white matter integrity analysis, and behavioral assays (rotarod and grip strength) in dMCAO, tMCAO, and perioperative stroke (PIS) models.

**Results:**

Using scRNA‐seq, we identified interferon‐induced transmembrane protein 3 (IFITM3) as a specific marker for a microglial subpopulation that was characterized by upregulated ACAT1, enhanced cholesterol esterification, and accumulation of cholesterol crystals and lipid droplets. This IFITM3^+^ microglia population peaked at 7 days post‐stroke and correlated with NLRP3 inflammasome activation and STING signaling. Pharmacological reduction of cholesterol burden with HβCD attenuated lipid droplet formation, suppressed mitochondrial DNA leakage, and inhibited STING pathway activation. Correspondingly, HβCD and C‐176 administration significantly reduced cerebral infarct size, mitigated white matter demyelination, and improved motor function in dMCAO and tMCAO models. We further found that AAV‐mediated STING knockdown recapitulated the above protective effects in HβCD and C‐176 treated stroke mice. Furthermore, HβCD treatment ameliorated microglial inflammation and improved functional outcomes in a PIS model.

**Conclusion:**

IFITM3^+^ microglia is a pro‐inflammatory and cholesterol‐laden subpopulation that exacerbates post‐stroke cerebral ischemic brain injury. Targeting the microglial cholesterol axis by HβCD or inhibiting the STING pathway represents a promising therapeutic strategy to mitigate ischemic brain injury and improve neurological function.

## Graphical abstract

After ischemic stroke, cholesterol accumulation in microglia is accompanied by the emergence of a pro‐inflammatory IFITM3^+^ subset with aberrant esterification and lipid droplet buildup. This metabolic stress is linked to mitochondrial dysfunction, STING activation, sustained neuroinflammation, and white matter injury. Cholesterol clearance by HβCD or STING inhibition attenuates inflammation and improves neurological recovery.

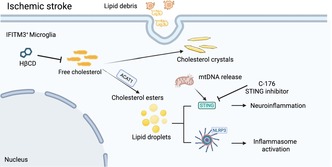

## 1 Introduction

The brain is the most cholesterol‐rich organ in the body [1, 2, 3, 4, 5], relying heavily on this lipid as an essential structural component of neuronal cell membranes. Within the central nervous system, cholesterol plays a pivotal role in maintaining myelin sheath integrity and facilitating proper neuronal signal transduction [6, 7, 8]. Consequently, impaired cholesterol metabolism has been broadly implicated in the pathogenesis of multiple neurodegenerative diseases, underscoring its central role in maintaining neurological health [6, 9, 10]. During cerebral ischemic stroke, the sudden breakdown of damaged neuronal tissue releases a massive burden of cholesterol and cellular debris into the microenvironment. This poses a severe challenge to resident brain microglia, which are tasked with phagocytosing cellular debris to modulate the local immune response and promote repair [11, 12, 13, 14, 15, 16]. Engulfing such lipid‐rich neuronal debris profoundly disrupts microglial intracellular cholesterol metabolism [17, 18]. However, the mechanisms regulating microglia cholesterol metabolism after cerebral ischemic stroke remain largely unknown.

Recent advances have begun to link intracellular metabolic stress directly to innate immune activation. Specifically, cellular cholesterol and lipid overload have been shown to trigger powerful inflammatory cascades, including the NLRP3 inflammasome [19], and cytosolic DNA sensing pathways such as the cGAS‐STING axis [20]. Activation of these sensors drives a robust type I interferon response, leading to the downstream expression of interferon‐stimulated genes, notably interferon‐induced transmembrane protein 3 (*Ifitm3*). While *Ifitm3* is known to regulate lipid raft composition and interferon signaling in other contexts [21], its potential role as a marker for metabolically stressed microglia during ischemic stroke remains entirely unexplored.

In this study, we demonstrate that cholesterol progressively accumulates in microglia following ischemic stroke, a process that is tightly associated with severe pro‐inflammatory activation. Utilizing single‐cell transcriptomics alongside extensive histological analyses, we identified a highly specific IFITM3^+^ microglia population defined by aberrant cholesterol esterification and lipid droplet accumulation. Furthermore, we demonstrate that pharmacological mobilization of cellular cholesterol using 2‐hydroxypropyl‐β‐cyclodextrin (HβCD) effectively reduces the burden of IFITM3^+^ lipid‐laden microglia, suppresses STING‐dependent neuroinflammation, and significantly improves overall neurological outcomes. Collectively, our findings identify IFITM3^+^ microglia as a critical pathogenic driver of post‐stroke inflammation and reveal that targeting cholesterol homeostasis represents a promising therapeutic strategy for ischemic stroke.

## 2 Methods

### 2.1 Animals

Adult male C57BL/6J mice (8–10 weeks) were obtained from Suzhou Cyagen Biosciences. All experiments were approved by the Institutional Animal Care and Use Committee of Cyagen Biosciences (Suzhou) Inc. (approval no. TACU23‐SY214) and performed according to the Institutional Guide for the Care and Use of Laboratory Animals. Animal data reporting followed the ARRIVE 2.0 guidelines [22] and STAIR X Stroke recommendations [23].

### 2.2 dMCAO (distal Middle Cerebral Artery Occlusion, Cerebral Ischemic Stroke Model)

Permanent focal cerebral ischemia was induced using the distal middle cerebral artery occlusion (dMCAO) model. Mice were anesthetized with 3% isoflurane for induction and maintained at 1.5% throughout the procedure. Core body temperature was monitored via a rectal probe and maintained at 37.0°C ± 0.5°C using a heating pad. The distal middle cerebral artery (MCA) was permanently occluded by electrocoagulation. Cerebral blood flow (CBF) was monitored using a laser speckle perfusion imaging system (PeriCam PSI System, Perimed) to confirm successful vessel occlusion [24].

### 2.3 tMCAO (transient Middle Cerebral Artery Occlusion, Cerebral Ischemic Stroke and Reperfusion Model)

Transient focal cerebral ischemia with reperfusion was induced using the intraluminal filament method. A 0.22 mm silicone‐coated filament was inserted via the external carotid artery into the internal carotid artery to occlude the origin of the MCA. Following 60 min of transient occlusion, the filament was retracted to restore CBF. Body temperature was maintained at 37.0°C ± 0.5°C throughout the procedure using a heating pad.

### 2.4 Perioperative Ischemic Stroke Model

To simulate perioperative stroke (PIS), in which major surgery‐induced injury precedes cerebral ischemia, mice underwent ileocolic bowel resection (ICR), followed 24 h later by dMCAO as described above [24, 25].

### 2.5 Pharmacological Interventions

Mice were randomly assigned to receive intraperitoneal (i.p.) injections. To promote cholesterol clearance, 2‐hydroxypropyl‐β‐cyclodextrin (HβCD; Sigma‐Aldrich, H107) was administered at a dose of 400 mg/kg, 2 h and 48 h after dMCAO induction. To modulate the STING pathway, the selective STING antagonist C‐176 (Selleck Chemicals, S6575) was administered at 10 mg/kg. Control groups received the corresponding vehicle: PBS for HβCD, or a mixture of 1% DMSO and corn oil for C‐176 and C‐176 + HβCD combinations [26, 27, 28].

### 2.6 Tissue Harvesting and Preparation

At the designated experimental endpoints, mice were deeply anesthetized via sodium pentobarbital and underwent transcardial perfusion with ice‐cold PBS to clear blood, followed by 4% paraformaldehyde (PFA) for fixation. Brains were carefully harvested and post‐fixed in 4% PFA for an additional 24 h (or 2–4 h for tracer experiments) at 4°C. For cryoprotection, brains were transferred to a 30% sucrose solution and stored at 4°C until they sank (approximately 48 h). Finally, the cryoprotected brains were embedded in Optimal Cutting Temperature (OCT) compound, frozen, and sectioned into 25–40 μm‐thick coronal slices using a cryostat.

### 2.7 Immunofluorescence Staining

Brain sections were permeabilized and blocked for 30 min at room temperature using a solution of 10% normal donkey serum in 1% Triton X‐100 in PBS (1% PBST). Subsequent washing steps were performed using 0.3% Triton X‐100 in PBS (0.3% PBST). Sections were then incubated overnight at 4°C with the following primary antibodies: anti‐Iba1 (Goat, 1:500, Abcam, ab5076; Rabbit, 1:500, Wako, 019‐19741), anti‐MAP2 (Rabbit, 1:500, Abcam, ab32454), anti‐SMI32 (NF‐H) (Mouse, 1:500, Biolegend, 801701), anti‐MBP (Rabbit, 1:200, Abcam, ab40390), anti‐NLRP3 (Rabbit, 1:500, Abcam, ab263899), anti‐PLIN2 (Rabbit, 1:500, Proteintech, 16215‐1‐AP), anti‐ACAT1 (Rabbit, 1:500, Abcam, ab154396), anti‐TOM20 (Rabbit, 1:500, Proteintech, 11802‐1‐AP), anti‐dsDNA (Rabbit, 1:500, Proteintech, 85781‐2‐RR), anti‐PLIN2 (Mouse, 1:500, Abcam, ab181463), and anti‐IFITM3 (Rabbit, 1:500, Proteintech, 11714–1‐AP). Following washes with 0.3% PBST, sections were incubated for 1 h with appropriate Alexa Fluor‐conjugated secondary antibodies (1:1000). Nuclei were counterstained with DAPI (0.5–1 μg/mL). For neutral lipid visualization, lipid droplets were stained using BODIPY 493/503 (Invitrogen, D3922). Free cholesterol was labeled with Filipin III (Sigma‐Aldrich, SAE0087). Images were acquired on an Olympus Fluoview FV3000 laser scanning confocal microscope. A blinded investigator performed all image analysis and quantification using ImageJ software (v1.52a, NIH).

### 2.8 Cholesterol Crystal Imaging

The intrinsic birefringence of cholesterol crystals was assessed using polarized light microscopy (PLM) [29]. Brain sections were examined under crossed polarizers with a rotating λ‐compensator plate on an Olympus FV3000 microscope. Crystal density was quantified using ImageJ software (v1.52a, NIH).

### 2.9 Luxol Fast Blue (LFB) Staining

To assess the integrity of myelin sheaths, brain sections were stained using a Luxol Fast Blue Myelin Stain Kit (Solarbio, G3245). Sections were incubated overnight in LFB solution, differentiated until gray/white matter contrast was distinct.

### 2.10 Single‐Cell RNA Sequencing

The scRNA‐seq experiment was conducted by professional researchers in the laboratory of NovelBio Bio‐Pharm Technology Co. Ltd. (Shanghai, China), following standard experimental protocols. For scRNA‐seq analysis, one mouse was included per experimental group (*n* = 1 per group). Peri‐infarct brain tissues were collected from the sham and dMCAO‐treated groups for single‐cell transcriptomic profiling. Mice were deeply anesthetized and transcardially perfused with ice cold phosphate buffered saline (PBS) to remove circulating blood before tissue collection. The brains were rapidly removed and placed in ice‐cold PBS, and peri‐infarct brain tissues were carefully dissected under a stereomicroscope to avoid contamination from non‐lesioned regions. The collected tissues were processed using the Adult Brain Dissociation Kit (Miltenyi Biotec) according to the manufacturer's instructions. Briefly, brain tissues were mechanically minced and subjected to enzymatic digestion at 37°C with gentle agitation. The resulting cell suspensions were gently triturated, filtered through cell strainers to remove debris and aggregates, and centrifuged at low speed. Red blood cells were lysed using a commercial red blood cell lysis buffer, and cells were washed and finally resuspended in PBS containing 0.04% BSA. Cell concentration and viability were assessed by dye exclusion, and only single‐cell suspensions with high viability were used for downstream single‐cell capture.

To profile the transcriptomes of individual brain‐derived single cells, the PanoCell‐ScRNA platform was used for transcriptomic information capture. A limited dilution approach was employed to randomly disperse the single‐cell suspension across more than 200,000 microwells, thereby achieving single‐cell capture. Beads functionalized with oligonucleotide barcodes were added in excess to reach saturation, thereby facilitating the pairing of one bead with one cell per microwell. Following lysis of the cells in the microwells, mRNA molecules were hybridized to the capture oligos conjugated to the surface of the beads. Beads were then pooled into one tube for reverse transcription and ExoI digestion. Upon completion of cDNA synthesis, each cDNA molecule received a unique molecular identifier (UMI) and a cell barcode at the 5′ end (matching the 3′ end of the mRNA transcript), enabling unambiguous assignment to its source cell. To generate whole‐transcriptome libraries, the PanoCell‐ScRNA platform's single‐cell whole‐transcriptome amplification (WTA) workflow was employed, including three key steps: random priming and extension (RPE), RPE amplification PCR, and WTA index PCR, to append Illumina sequencing adapters and unique sample indices. Agilent High Sensitivity DNA Chip (Bioanalyzer system) and Thermo Fisher Scientific Qubit High Sensitivity DNA Assay were used for library quantification. Qualified libraries were then pooled and sequenced on the BGI sequencing platform with 150‐bp paired‐end reads.

### 2.11 scRNA‐Seq Statistical Analysis

Raw sequencing data were processed using fastp with its default parameter settings to trim adapter sequences and exclude low‐quality reads, yielding clean sequencing reads [30]. UMI‐tools was utilized in single‐cell transcriptome analysis to establish the cell barcode whitelist [31]. Clean UMI‐filtered reads were mapped to the mouse genome (mm10, Ensembl version 91) via STAR, with tailored parameters from the UMI‐tools standard pipeline to determine each sample's UMI counts. To alleviate inter‐sample heterogeneity stemming from variations in sequencing depth, down‐sampling of raw sequencing reads was performed using the average reads per cell of each sample as a reference, producing a unified and standardized cell expression matrix with sample‐specific barcodes.

To ensure data reliability, cells were subjected to quality filtering: only those expressing more than 200 genes and exhibiting a mitochondrial UMI proportion of less than 30% were retained for downstream analysis. After quality control, a total of 15,942 cells were retained for downstream analysis. The Seurat R package (v4.0.6) (https://satijalab.org/Seurat/) was used for cell normalization, scaling, and regression based on the UMI count matrix and mitochondrial UMI percentage. We used Seurat's regression‐based correction strategy to regress out the effects of library size (total UMI counts) and mitochondrial percentage (MT%). Because all samples were processed in parallel under identical experimental conditions and sequenced in the same run, no evident experimental batch structure was identified; therefore, no batch correction was applied. Technical covariates, including total UMI counts and mitochondrial UMI percentage, were regressed out during Seurat preprocessing.

Highly variable genes were identified from the normalized data and used for principal component analysis (PCA) and subsequent downstream analyses. For nonlinear dimensionality reduction and visualization, we used Uniform Manifold Approximation and Projection (UMAP) based on the top principal components to obtain a low‐dimensional embedding of the single‐cell transcriptomic landscape.

Unsupervised cell clustering was performed using a graph‐based clustering method implemented in Seurat on the PCA‐based shared nearest‐neighbor graph (resolution = 0.8), yielding distinct cell clusters. To characterize cluster‐specific marker genes, the Wilcoxon rank‐sum test was utilized in combination with the FindAllMarkers (for multiple‐cluster comparisons) and FindMarkers (for pairwise comparisons) functions, with selection based on three key criteria: log fold change > 0.25, *p* < 0.05, and min.pct > 0.1. Cluster‐specific marker genes identified by these analyses were used to annotate each cluster to known cell types and functional states based on canonical markers and reference to published single‐cell RNA‐seq datasets.

### 2.12 Transmission Electron Microscopy (TEM)

Samples of peri‐infarct cortex and white matter were fixed in 2.5% glutaraldehyde, post‐fixed in 1% osmium tetroxide with 1.5% potassium ferricyanide, and dehydrated through a graded ethanol series. Ultrathin sections (60 nm) were cut and stained with uranyl acetate and lead citrate for ultrastructural analysis.

### 2.13 Behavioral Test

Latency to fall or spin around on the rungs was measured following placement of mice on a 3.0 cm diameter rotating rod (YLS‐4D) and training to maintain position for 300 s under acceleration from 5 to 40 rpm.

Grip strength was measured to evaluate neuromuscular function. Following forelimb placement on the metal bar, gentle tail traction was applied to elicit maximal grip resistance, with peak values automatically logged. Data represent the mean of three replicate trials per mouse. All behavioral assessments were performed by investigators blinded to group allocation. Sample sizes for each group are indicated in the corresponding figure legends. Power calculations were not performed a priori; however, group sizes were chosen based on our previous studies and commonly used standards in the field to ensure adequate statistical sensitivity.

### 2.14 In Vivo Microglia‐Targeted STING Knockdown Using AAV9 Vectors

In vivo STING knockdown was achieved using recombinant adeno‐associated virus serotype 9 (AAV9) vectors. A microglia‐targeted STING knockdown virus, AAV9‐F4/80‐miR30‐shRNA (STING), and a corresponding negative control virus, AAV9‐F4/80‐miR30‐shRNA (scramble), were constructed and packaged by WZ Biosciences Inc. (China). The shRNA targeting sequence for mouse STING was 5′‐CAACATTCGATTCCGAGATAT‐3′. For simplicity, these viruses are hereafter referred to as AAV‐shSTING and AAV‐shNC, respectively. Purified AAV9 vectors were diluted in sterile phosphate‐buffered saline (PBS) to a working concentration (~1–5 × 10^12^ genome copies/mL). For intracerebroventricular (ICV) injection, mice were anesthetized and placed in a stereotaxic apparatus. AAV‐shSTING or AAV‐shNC (2–5 μL per mouse) was injected into the lateral ventricle at the following coordinates relative to bregma: anteroposterior (AP), −0.3 mm; mediolateral (ML), ±1.0 mm; dorsoventral (DV), −2.5 mm. The injection rate was 0.2–0.5 μL/min. After injection, the needle was left in place for 5 min before slow withdrawal to minimize reflux. Mice were allowed to recover for 3 weeks to ensure sufficient viral expression before subsequent dMCAO surgery.

### 2.15 Statistical Analysis

Statistical tests were conducted using GraphPad Prism software (v9). Data are mean ± SD. For comparisons between two experimental groups, a two‐tailed unpaired Student's *t*‐test was employed. For analyses involving multiple groups, a one‐way or two‐way analysis of variance (ANOVA) was used, followed by Bonferroni post hoc correction for multiple comparisons. A *p* value < 0.05 was deemed statistically significant. Partial bioinformatics analyses were conducted using the NovelBrain Cloud Analysis Platform (www.novelbrain.com).

## 3 Results

### 3.1 Cholesterol Accumulates in Pro‐Inflammatory Microglia After Cerebral Ischemic Stroke

**FIGURE 1:**
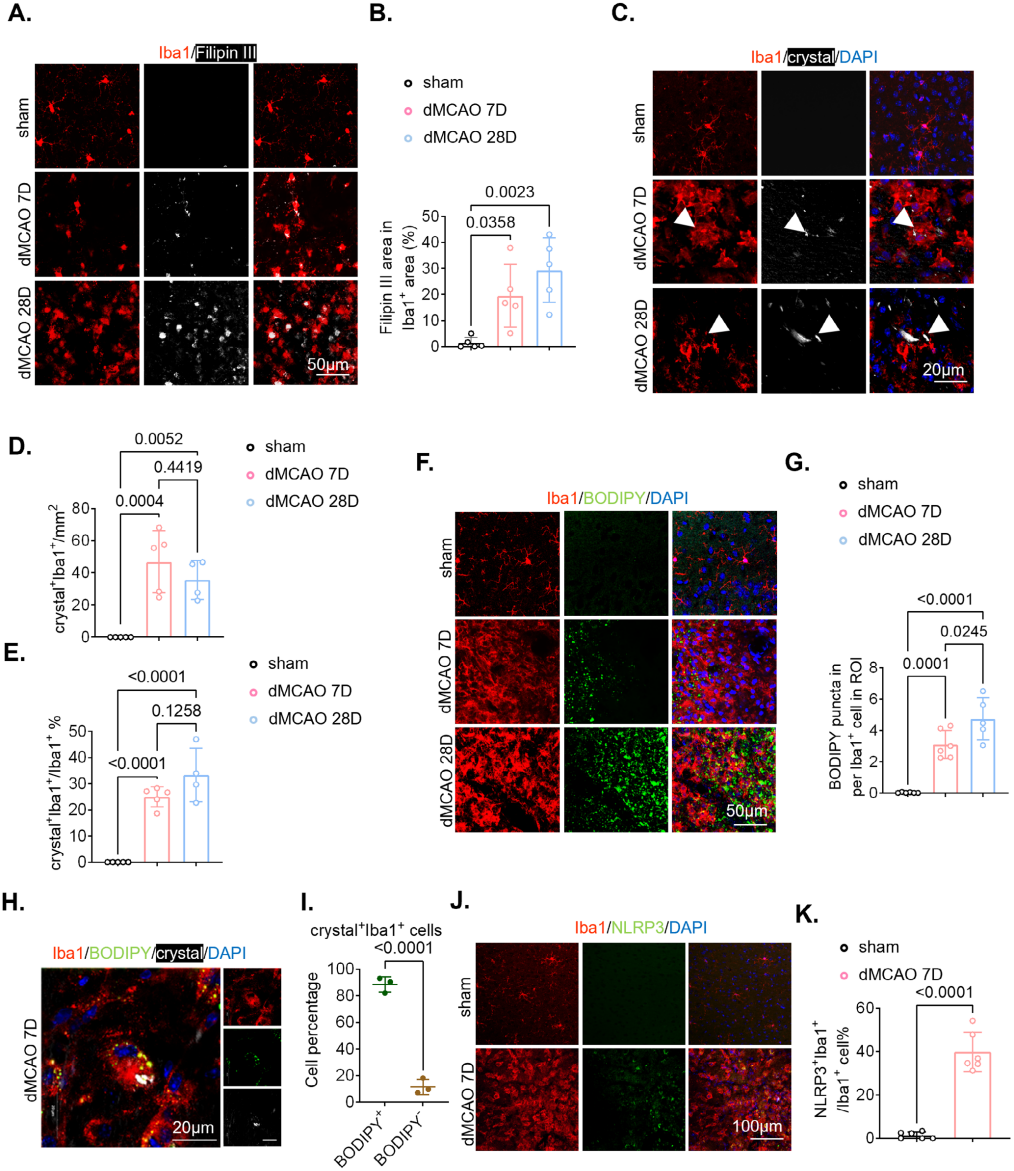
Microglia display cholesterol metabolic reprogramming and neuroinflammation after ischemic stroke. (A–B) Immunofluorescence analysis of free cholesterol in microglia within the peri‐infarct region in sham, dMCAO 7D, and dMCAO 28D groups. (A) Representative images showing Filipin III staining in Iba1^+^ cells. Scale bar = 50 μm. (B) Quantification of Filipin III area in Iba1^+^ cells (*n* = 5 per group one‐way ANOVA with Bonferroni post hoc correction). (C–E) Immunofluorescence analysis of cholesterol crystals in microglia in the peri‐infarct region. (C) Representative images of crystal^+^Iba1^+^ cells in sham, dMCAO 7D, and dMCAO 28D groups. (D) Quantification of the proportion of crystal^+^Iba1^+^ cells. (E) Quantification of the absolute number of crystals^+^Iba1^+^ cells (*n* = 4–5 per group, one‐way ANOVA with Bonferroni post hoc correction). Scale bar = 20 μm. (F, G) Immunofluorescence analysis of lipid droplets in microglia after ischemic stroke. (F) Representative images showing Iba1^+^BODIPY^+^ cells in sham, dMCAO 7D, and dMCAO 28D groups. (G) Quantification of Iba1^+^BODIPY^+^ cells (*n* = 5–6 per group, one‐way ANOVA with Bonferroni post hoc correction). Scale bar = 50 μm. (H, I) Colocalization of cholesterol crystals and lipid droplets in microglia in the dMCAO 7D group. (H) Representative immunofluorescence images of Iba1 and BODIPY with bright field polarized light imaging of crystals from the dMCAO mice 7 days after stroke. (I) Quantification of crystal^+^ cell percentage in BODIPY^+^ versus BODIPY^‐^ in Iba1^+^ populations (unpaired *t*‐test). Scale bar = 20 μm. (J, K) Representative immunofluorescence images (J) and quantification (K) of Iba1^+^NLRP3^+^ cells in the peri‐infarct region in sham and dMCAO 7D groups (*n* = 6 per group, unpaired *t*‐test). Scale bar = 100 μm.

To investigate the temporal dynamics of cholesterol accumulation in microglia following distal middle cerebral artery occlusion (dMCAO), we quantified free cholesterol levels, cholesterol crystals, and neutral lipid droplets in Iba1^+^ cells within the peri‐infarct cortex. Filipin III staining revealed a progressive accumulation of free cholesterol in microglia from the sham condition through 7 and 28 days post‐dMCAO (Figure 1A,B). Concurrently, polarized light microscopy (PLM) demonstrated a parallel temporal increase in cholesterol crystal deposition within these cells, with the proportion of crystal‐laden Iba1^+^ microglia peaking at day 7 post‐injury (Figure 1C–E). Furthermore, BODIPY staining confirmed a significant elevation of neutral lipid droplets in microglia at 7 days post‐dMCAO compared to sham controls (Figure 1F,G). Notably, PLM combined with immunofluorescence analyses revealed that approximately 90% of the microglia harboring cholesterol crystals were also enriched with BODIPY^+^ lipid droplets (Figure 1H,I). This severe lipid overload was accompanied by a marked upregulation of NLRP3 expression in these microglia (Figure 1J,K). Collectively, these findings indicate that cerebral ischemia induces a progressive, pathological accumulation of cholesterol crystals and lipid droplets in microglia, which associates with an augmented pro‐inflammatory state.

### 3.2 *Ifitm3* Identifies Microglia With Enhanced Cholesterol Esterification and Lipid

#### 3.2.1 Accumulation Following Ischemic Stroke

**FIGURE 2:**
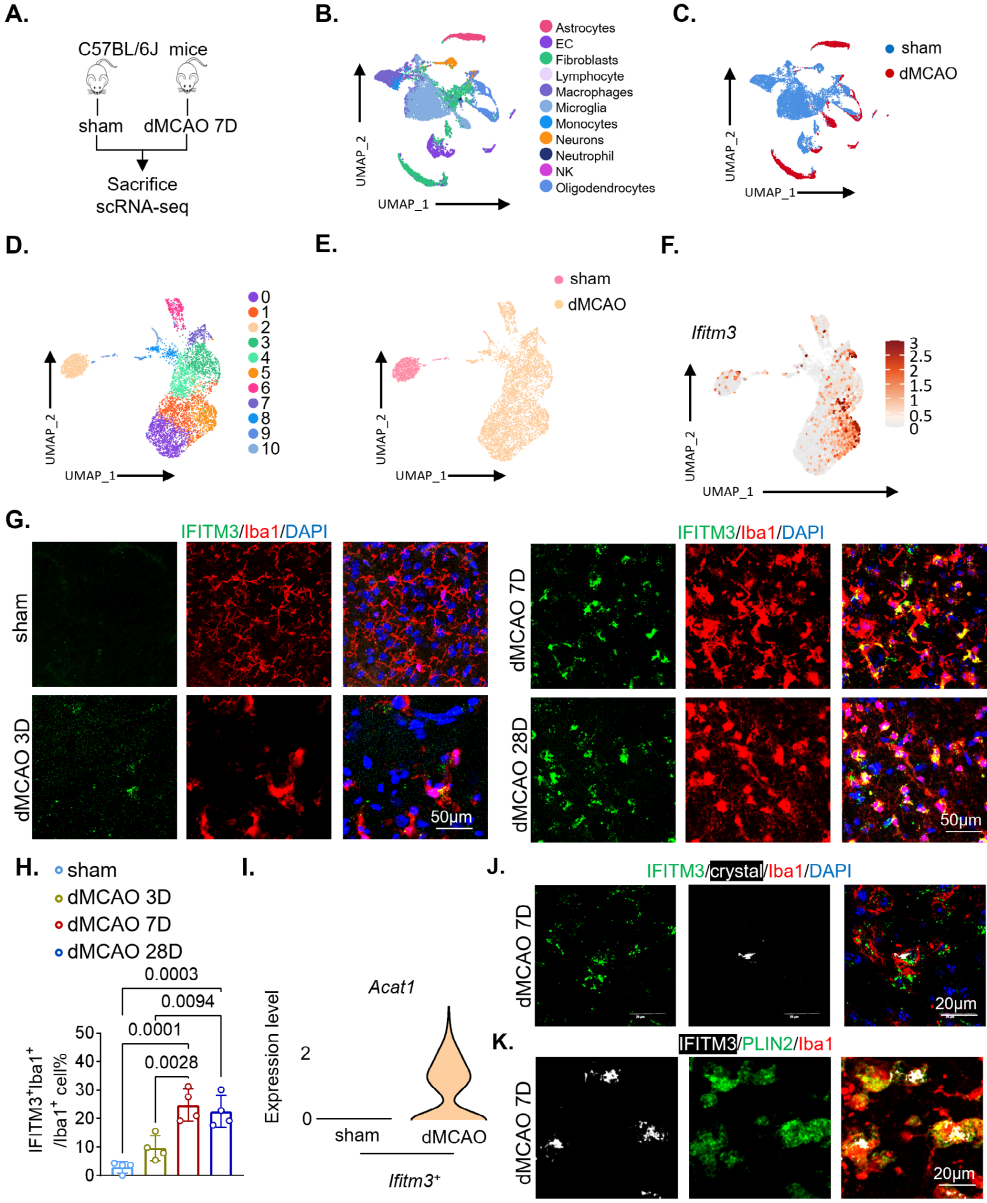
IFITM3^*+*^ microglia exhibit elevated cholesterol esterification following ischemic stroke. (A) Schematic of the experimental design. (B) UMAP showing cells in the peri‑infarct cortex from sham and 7 days post‐dMCAO groups. (C) UMAP visualization showing sample group distribution across all cell clusters. (D) Reclustering of microglia identifying distinct microglia subpopulations from sham and 7 days post‑dMCAO groups. (E) UMAP visualization showing sample group distribution across microglia. (F) UMAP plots showing *Ifitm3* expression across microglia from sham and 7 days post‐dMCAO. (G–H) Representative immunofluorescence images (G) and quantification (H) of IFITM3^+^ Iba1^+^ cells in the peri‐infarct region in sham and at 3, 7, and 28 days post‐dMCAO (*n* = 4 per group, one‐way ANOVA, Bonferroni post hoc correction). Scale bar = 50 μm. (I) Violin plot showing *Acat1* mRNA expression levels in *Ifitm3* ^*+*^ microglia subpopulations from sham and dMCAO 7D groups. (J) Representative immunofluorescence images showing Iba1 and IFITM3 staining combined with polarized light imaging of cholesterol crystals in the peri‐infarct region at 7 days post dMCAO. Scale bar = 20 μm. (K) Representative immunofluorescence images showing IFITM3, PLIN2, and Iba1 staining in the peri‐infarct region at 7 days post‐dMCAO. Scale bar = 20 μm.

To elucidate the molecular mechanisms regulating microglia cholesterol homeostasis after stroke, we performed single‐cell RNA sequencing (scRNA‐seq) on cells from the ischemic hemisphere of sham and dMCAO mice (Figure 2A). We identified major immune populations (Figure 2B,C) and reclustered microglia based on canonical markers (*Cx3cr1, P2ry12, Hexb*) [32] (Figure 2D,E, Figure S1A,B). By examining gene expression patterns across microglia subpopulations, we found prominent upregulation of *Ifitm3* in specific clusters following ischemic stroke (Figure 2F). Immunofluorescence time‐course analysis further validated this transcriptomic shift, demonstrating that the proportion of IFITM3^+^Iba1^+^ microglia in the peri‐infarct region surged by day 3, peaked at day 7, and remained persistently elevated up to 28 days post‐dMCAO (Figure 2G,H). Transcriptional profiling of this *Ifitm3* ^+^ subset highlighted a robust induction of *Acat1*, a critical enzyme responsible for cholesterol esterification and subsequent lipid droplet storage [33] (Figure 2I). Consistent with these scRNA‐seq findings, triple immunostaining combined with PLM confirmed that IFITM3^+^ microglia were specifically laden with both cholesterol crystals and PLIN2^+^ neutral lipid droplets at 7 days post‐injury (Figure 2J,K). These data delineate Ifitm3 as a robust marker for a transcriptionally distinct microglial state characterized by aberrant cholesterol esterification and profound lipid overload following stroke.

### 3.3 HβCD Reduces IFITM3 ^+^ Microglia Burden and Attenuates Cholesterol Esterification Following Ischemic Stroke

**FIGURE 3:**
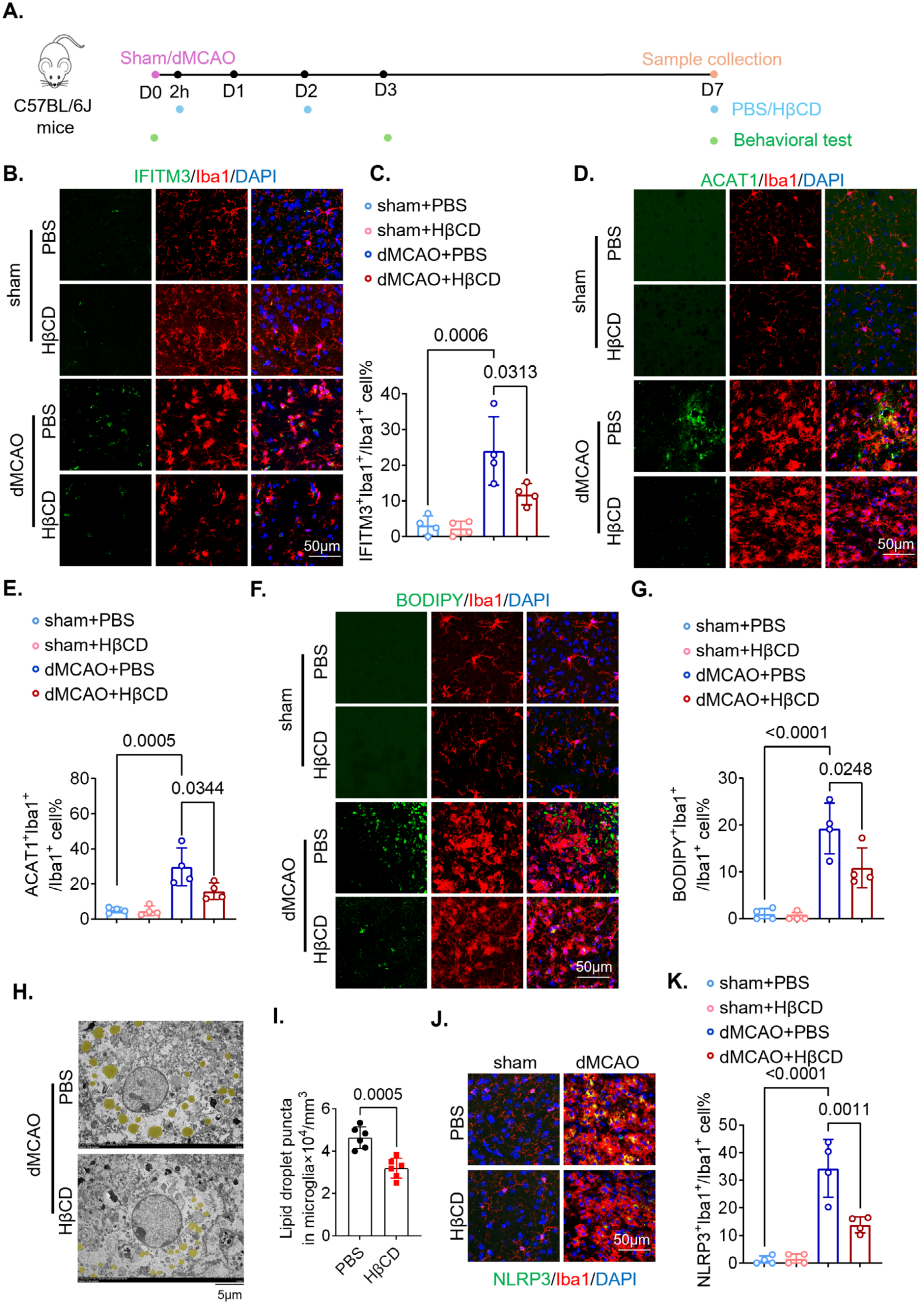
HβCD treatment attenuates cholesterol esterification and neuroinflammation in microglia following stroke. (A) Schematic illustration of the experimental design. C57BL/6J mice were subjected to sham or dMCAO surgery on day 0. HβCD or PBS control was administered via intraperitoneal injection at 2 and 48 h post‐surgery. Behavioral assessments were performed at baseline, day 3 (D3), and day 7 (D7). Animals were killed at day 7 for sample collection and subsequent analyses. (B, C) Representative immunofluorescence images (B) and quantification (C) of IFITM3^+^Iba1^+^ cells in the peri‐infarct region in sham+PBS, sham+HβCD, dMCAO+PBS, and dMCAO+HβCD groups (*n* = 4 per group, one‐way ANOVA with Bonferroni post hoc correction). Scale bar = 50 μm. (D, E) Representative immunofluorescence images (D) and quantification (E) of ACAT1^+^Iba1^+^ cells in the peri‐infarct region in sham+PBS, sham+HβCD, dMCAO+PBS, and dMCAO+HβCD groups (*n* = 4 per group, one‐way ANOVA with Bonferroni post hoc correction). Scale bar = 50 μm. (F, G) Representative immunofluorescence images (F) and quantification (G) of BODIPY^+^Iba1^+^ cells in the peri‐infarct region in sham+PBS, sham+HβCD, dMCAO+PBS, and dMCAO+HβCD groups (*n* = 4 per group, one‐way ANOVA with Bonferroni post hoc correction). Scale bar = 50 μm. (H, I) Representative transmission electron microscopy images (H) and quantification (I) of lipid droplets in microglia in the peri‐infarct cortex at 7 days post dMCAO in PBS and HβCD‐treated mice (*n* = 6 per group, unpaired *t*‐test). Scale bar = 5 μm. Yellow circle: lipid droplets. (J, K) Representative immunofluorescence images (J) and quantification (K) of NLRP3^+^Iba1^+^ cells in the peri‐infarct region in sham+PBS, sham+HβCD, dMCAO+PBS, and dMCAO+HβCD groups (*n* = 4 per group, one‐way ANOVA with Bonferroni post hoc correction). Scale bar = 50 μm.

We next sought to determine whether pharmacological mobilization of cellular cholesterol could mitigate this pathological microglial state. Mice were systemically administered 2‐hydroxypropyl‐β‐cyclodextrin (HβCD), a well‐characterized cholesterol‐depleting agent, at 2 and 48 h post‐dMCAO [28] (Figure 3A). Filipin III staining at day 7 confirmed that HβCD treatment robustly attenuated the accumulation of free cholesterol within Iba1^+^ microglia in the peri‐infarct region compared to vehicle‐treated controls (Figure S2A,B). Importantly, HβCD administration significantly diminished the overall burden of IFITM3^+^Iba1^+^ microglia (Figure 3B,C). This reduction coincided with a decrease in the proportion of ACAT1^+^Iba1^+^ microglia, indicating an effective suppression of aberrant cholesterol esterification (Figure 3D,E). Consequently, both BODIPY staining and transmission electron microscopy (TEM) independently verified a substantial reduction in intracellular lipid droplet accumulation within microglia following HβCD therapy (Figure 3F–I). This metabolic correction translated to a dampening of neuroinflammation, as evidenced by significantly reduced numbers of NLRP3‐expressing microglia (Figure 3J,K). Thus, facilitating cholesterol mobilization via HβCD effectively curtails lipid droplet formation and blunts the pro‐inflammatory activation of IFITM3^+^ microglia.

### 3.4 HβCD‐Mediated Cholesterol Clearance Alleviates Ischemic Brain Injury and Promotes Functional Recovery

**FIGURE 4:**
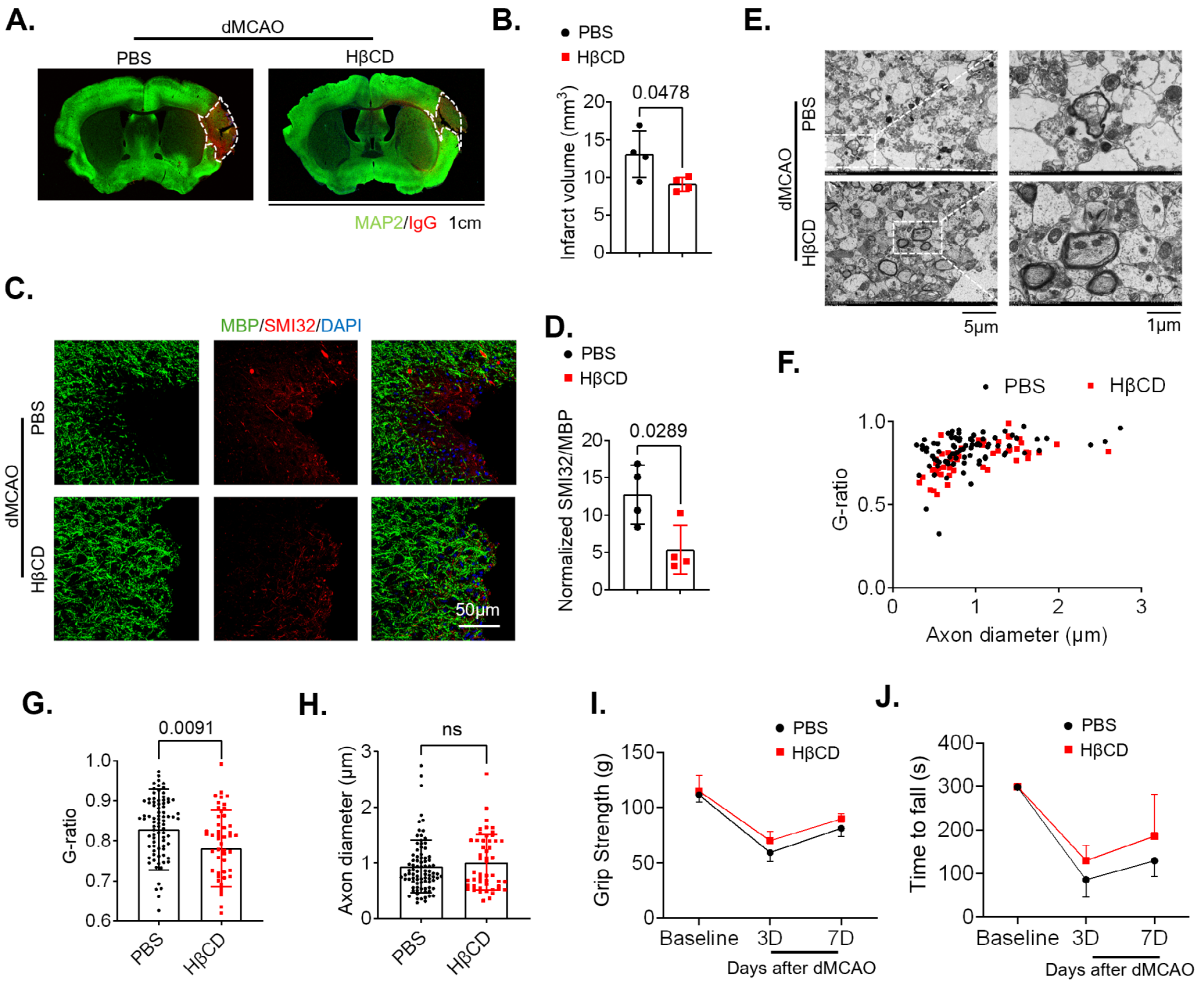
HβCD treatment reduces brain injury and improves functional recovery after ischemic stroke. (A, B) Representative MAP2 and IgG immunofluorescence images (**A**) and quantification (B) of infarct areas in PBS and HβCD‐treated groups at 7 days post dMCAO (*n* = 4 per group, unpaired *t*‐test). Scale bar = 1 cm. (C, D) Representative immunofluorescence images of SMI32 and MBP staining (C) and normalized SMI32 to MBP ratio (D) of the peri‐infarct cortex in PBS and HβCD‐treated groups at 7 days post dMCAO (*n* = 4 per group, unpaired *t*‐test). Scale bar = 50 μm. (E) Representative transmission electron microscopy images of myelinated axons in the peri‐infarct cortex at 7 days post dMCAO (left panel scale bar = 5 μm, right panel scale bar = 1 μm). (F) G‐ratio analysis of remyelinated axons plotted against axonal diameter. (G) Scatter plot showing overall G‐ratio in HβCD‐treated mice compared to PBS‐treated mice (unpaired *t*‐test). (H) Analysis of axonal diameters between PBS and HβCD‐treated groups (unpaired *t*‐test). (I) Grip strength test performed at baseline, day 3, and day 7 post dMCAO in PBS and HβCD‐treated groups (*n* = 4 per group, two‐way ANOVA with Bonferroni post hoc correction). (J) Rotarod test measuring latency to fall at baseline, day 3, and day 7 post dMCAO (*n* = 4 per group, two‐way ANOVA with Bonferroni post hoc correction).

Given the capacity of HβCD to alleviate microglial metabolic stress, we next evaluated its broader impact on ischemic brain injury and functional restoration. At 7 days post‐dMCAO, HβCD‐treated mice exhibited significantly reduced infarct volumes compared to the vehicle cohort, as delineated by MAP2 and IgG immunostaining (Figure 4A,B). Furthermore, co‐labeling for SMI32 and MBP revealed an attenuated SMI32/MBP ratio in the peri‐infarct cortex, indicative of robust protection against ischemia‐induced white matter degradation (Figure 4C,D). TEM analysis corroborated this structural preservation, showing a significantly reduced G‐ratio in myelinated axons of HβCD‐treated mice, without altering overall axonal diameter distributions (Figure 4E–H). HβCD administration significantly improved motor deficits, evidenced by enhanced grip strength and prolonged rotarod latency at both 3 and 7 days post‐injury (Figure 4I,J). Crucially, the protective efficacy of HβCD was independently validated in a tMCAO reperfusion model, which similarly yielded diminished infarct sizes (Figure S4A,B), preserved myelin integrity via Luxol Fast Blue staining, and reduced SMI32/MBP ratios across multiple brain regions including the cortex, striatum, and external capsule (Figure S4C,D). Together, these results highlight the therapeutic viability of reducing microglial cholesterol burden to rescue white matter integrity and neurological function after stroke.

### 3.5 Therapeutic Suppression of STING Recapitulates HβCD Mediated Neuroprotection

**FIGURE 5:**
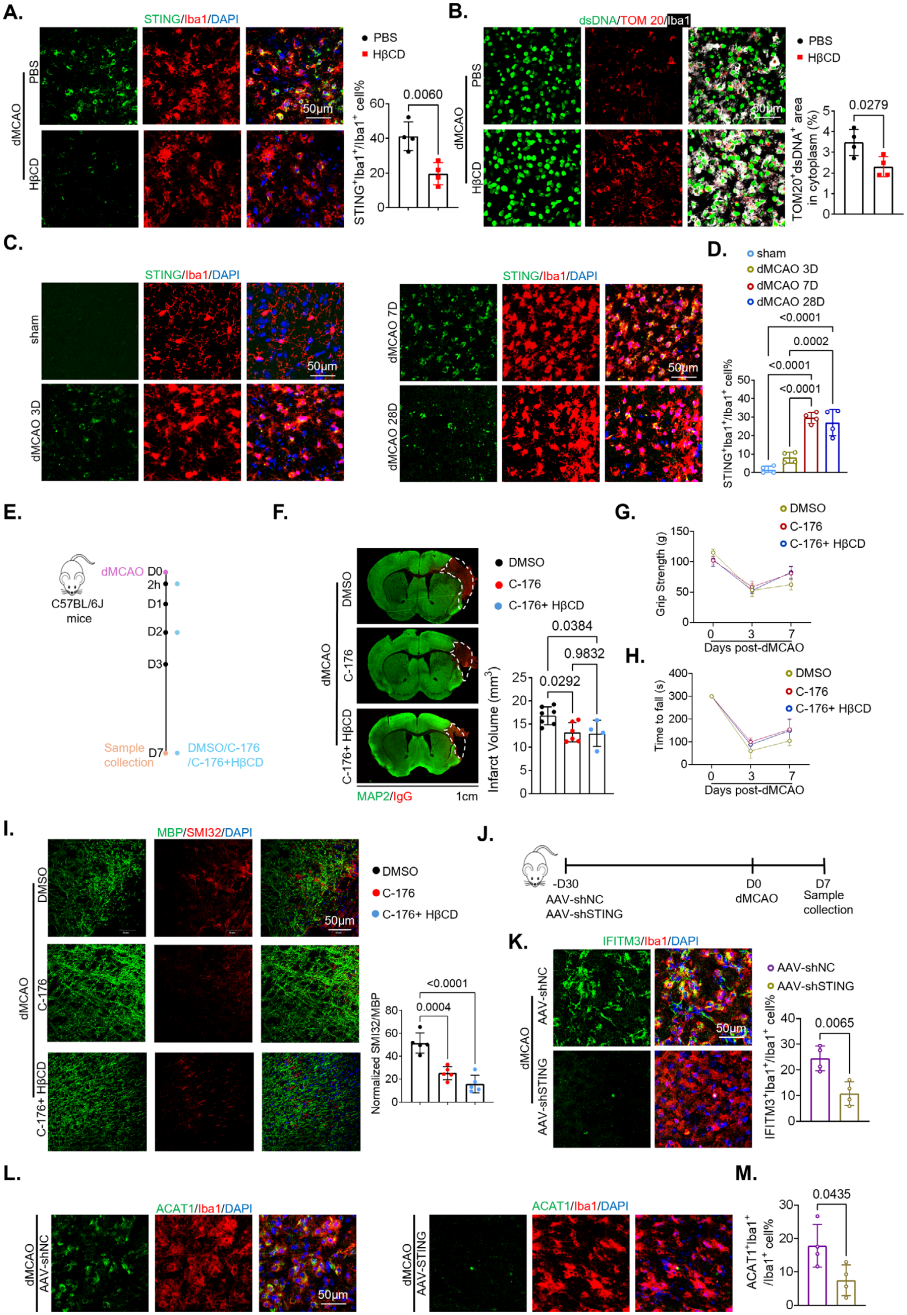
C‐176 reduces infarct volume and demyelination by suppressing STING. (A) Representative immunofluorescence images and quantitative analysis of STING^+^Iba1^+^ cells in the peri‐infarct cortex at 7 days post dMCAO in PBS and HβCD‐treated mice. Scale bars = 50 μm (*n* = 4 per group, unpaired *t*‐test). (B) Representative immunofluorescence images and quantification of TOM20^+^dsDNA^+^ area within Iba1^+^ cytoplasm in the peri‐infarct region at 7 days post dMCAO in PBS and HβCD‐treated mice. Scale bars = 50 μm (*n* = 4 per group, unpaired *t*‐test). (C, D) Representative immunofluorescence images (C) and quantification (D) of STING^+^ Iba1^+^ cells in the peri‐infarct region in sham 3, 7, and 28 days post dMCAO (*n* = 6 per group, one‐way ANOVA, Bonferroni post hoc correction). Scale bar = 50 μm. (E) Schematic of the experimental design. C57BL/6J mice were subjected to dMCAO surgery on day 0. Mice received intraperitoneal injections of DMSO, C‐176, or C‐176+HβCD at 2 and 48 h post‐surgery. All animals were killed at day 7 for tissue collection and subsequent analyses. (F) Representative MAP2 and IgG immunofluorescence images and quantification of infarct areas in DMSO, C‐176, and C‐176+HβCD‐treated mice at 7 days post dMCAO (*n* = 4–7 per group, one‐way ANOVA, Bonferroni post hoc correction). Scale bar = 1 cm. (G) Grip strength test performed at baseline, day 3, and day 7 in DMSO, C‐176, and C‐176+HβCD‐treated mice at 7 days post dMCAO (*n* = 4 per group, two‐way ANOVA with Bonferroni post hoc correction). (H) Rotarod test measuring latency to fall at baseline, day 3, and day 7 in DMSO, C‐176, and C‐176+HβCD‐treated mice at 7 days post dMCAO (*n* = 4 per group, two‐way ANOVA with Bonferroni post hoc correction). (I) Representative immunofluorescence images of SMI32 and MBP staining and normalized SMI32 to MBP ratio of the peri‐infarct cortex in DMSO, C‐176, and C‐176+HβCD‐treated mice at 7 days post dMCAO (*n* = 4–5 per group, one‐way ANOVA with Bonferroni post hoc correction). Scale bar = 50 μm. (J) Schematic of the experimental design. (K) Representative immunofluorescence images and quantification of IFITM3^+^Iba1^+^ cell in the peri‐infarct region in AAV‐shNC and AAV‐shSTING groups (*n* = 4 per group, unpaired *t*‐test). Scale bar = 50 μm. (L‐M) Representative immunofluorescence images (L) and quantification (M) of ACAT1^+^Iba1^+^ cells in the peri‐infarct region in AAV‐shNC and AAV‐shSTING groups (*n* = 4 per group, unpaired *t*‐test). Scale bar = 50 μm.

Excessive lipid accumulation is known to provoke innate immune sensors, particularly the cGAS‐STING pathway, which potently induces interferon‐stimulated genes such as *Ifitm3*. Immunofluorescence analysis at 7 days post‐dMCAO revealed a prominent surge in STING^+^Iba1^+^ microglia, a response that was profoundly suppressed by HβCD treatment (Figure 5A). To assess STING expression in peripherally derived macrophages, Ms4a3‐Ai14 mice were subjected to MCAO and analyzed at 7 days post‐injury. STING exhibited minimal colocalization with tdTomato^+^ cells in the peri‐infarct region, with only 7.04% of the total STING signal area overlapping with tdTomato (Figure S3A,B). To explore the upstream triggers linking cholesterol to STING activation, we assessed mitochondrial integrity. We found that HβCD significantly restrained mitochondrial DNA (mtDNA) leakage into the cytosol, as quantified by decreased cytosolic TOM20^+^ dsDNA^+^ colocalization in microglia (Figure 5B). To test whether STING signaling acts as the primary downstream effector of cholesterol‐induced injury, we administered the selective STING antagonist C‐176. Temporal profiling confirmed that microglial STING expression peaks at day 7 and remains sustained up to day 28 post‐ischemia (Figure 5C,D). Strikingly, C‐176 treatment significantly restricted infarct expansion while the combination of C‐176 and HβCD showed no additional reduction compared to C‐176 alone (Figure 5E,F). This non‐additive effect was mirrored in behavioral assays, where C‐176 robustly improved grip strength and rotarod performance, with no further functional gains observed in the dual‐treatment group (Figure 5G,H). We also found that C‐176 significantly decreased the normalized SMI32/MBP ratio compared to DMSO‐treated mice, and the addition of HβCD did not further enhance this protective effect (Figure 5I). To circumvent potential off‐target pharmacological effects, we utilized AAV9‐mediated delivery of shRNA to specifically knock down STING in microglia in vivo prior to dMCAO. Genetic silencing of STING not only reduced the abundance of IFITM3^+^ microglia but also downregulated ACAT1 expression in the peri‐infarct region (Figure 5J–M). Collectively, these findings suggest that STING signaling is an important downstream mediator linking microglial cholesterol dysregulation to neuroinflammation, white matter injury, and functional deficits after ischemic stroke.

### 3.6 HβCD Treatment Ameliorates Neuroinflammation and Improves Outcomes Following PIS

**FIGURE 6:**
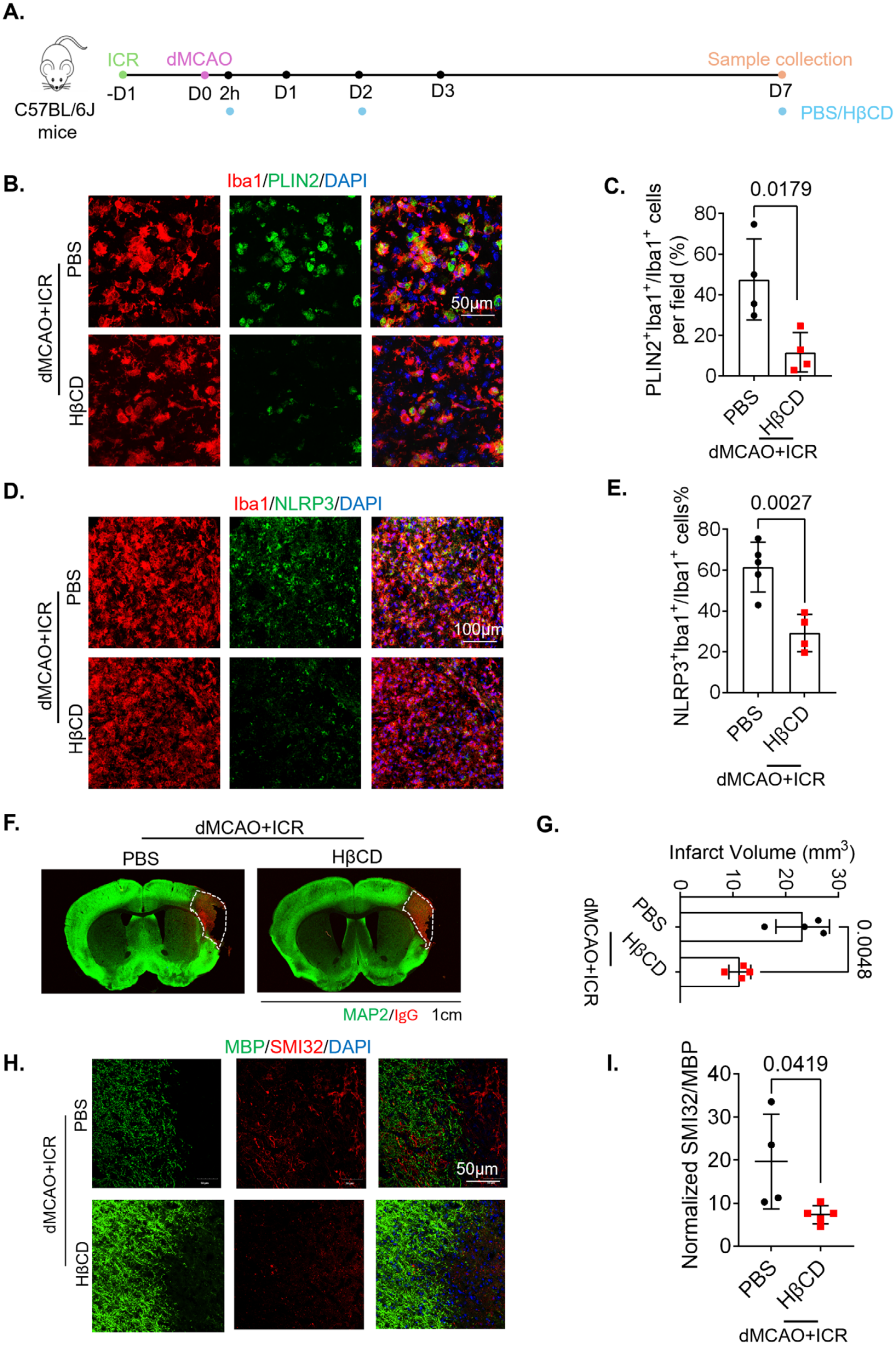
HβCD treatment ameliorates neuroinflammation and improves PIS outcomes. (A) Schematic of the experimental design. (B, C) Representative immunofluorescence images (B) and quantification (C) of Iba1^+^PLIN2^+^ cells in the peri‐infarct region in PBS‐ and HβCD‐treated mice in perioperative stroke groups (*n* = 4 per group, unpaired *t*‐test). Scale bar = 50 μm. (D, E) Representative immunofluorescence images (D) and quantification (E) of Iba1^+^NLRP3^+^ cells in the peri‐infarct region in PBS and HβCD‐treated mice in perioperative stroke groups (*n* = 4 per group, unpaired *t*‐test). Scale bar = 100 μm. (F, G) MAP2/IgG immunofluorescence staining (F) and quantification (G) of infarct volume at 7 days post‐dMCAO in PBS and HβCD‐treated mice in perioperative stroke groups (*n* = 4 per group, unpaired *t*‐test). Scale bar = 1 cm. (H, I) Immunofluorescence analysis of the normalized SMI32/MBP ratio in the peri‐infarct cortex at 7 days post‐dMCAO in PBS‐ and HβCD‐treated mice in perioperative stroke groups (*n* = 4–5 per group, unpaired *t*‐test). Scale bar = 50 μm.

Perioperative stroke (PIS) is a devastating complication of surgery with increased mortality and morbidity [34, 35]. To determine the translational relevance of targeting cholesterol metabolism in this aggravated context, we utilized a well‐established PIS model comprising ileocolic bowel resection (ICR) followed 24 h later by dMCAO [24, 25](Figure 6A). When HβCD was administered post‐surgery, we observed a dramatic reduction in the density of PLIN2^+^ lipid‐laden microglia and a concurrent decline in NLRP3^+^ microglial populations compared to vehicle‐treated PIS mice (Figure 6B–E). This potent anti‐inflammatory effect yielded significant tissue protection, manifesting as markedly reduced cerebral infarct volumes at 7 days post‐ischemia (Figure 6F,G). Moreover, HβCD successfully ameliorated white matter degradation, as demonstrated by a normalized SMI32/MBP ratio in the peri‐infarct cortex (Figure 6H,I). Collectively, these findings underscore that targeted cholesterol mobilization effectively curtails microglial lipid toxicity and mitigates the compounded neurological damage characteristic of perioperative stroke.

## 4 Discussion

In this study, we identified IFITM3^+^ microglia as a cholesterol‐laden microglial subpopulation characterized by aberrant cholesterol esterification, lipid droplet accumulation, and cholesterol crystal deposition after cerebral ischemic stroke. Our data suggest that this population is associated with mitochondrial dysfunction, STING pathway activation, and sustained neuroinflammation. These findings support the possibility that targeting microglial cholesterol clearance and STING signaling may represent a therapeutic strategy for ischemic stroke and PIS.

The brain undergoes profound lipid dysregulation following ischemic injury [4, 36]. The breakdown of cellular membranes releases a massive amount of cholesterol that requires clearance by phagocytes [6, 37, 38]. In cases of large brain infarcts or failed recanalization, the burden of cellular debris is particularly severe [39, 40], leading to exacerbated neuroinflammation. Ceramides derived from astrocytes exacerbate cerebral ischemic injury by activating cGAS/STING signaling via oxidative stress‐mediated mitochondrial dysfunction [41]. In ischemic brain injury, TREM2 regulates microglial lipid metabolism and activates the TGF‐β1/Smad2/3 pathway to suppress neuroinflammation, protect neurons, and alleviate ischemic stroke injury [42]. Persistent microglial activation causes cholesterol buildup to sustain post‐stroke neuroinflammation, and activating CYP46A1 relieves cholesterol overload to boost brain repair and functional recovery [29]. However, the specific microglial subpopulations associated with post‐stroke cholesterol dysregulation remain incompletely defined.

Our scRNA‐seq analysis identified *Ifitm3* as a molecular marker defining microglia with enhanced cholesterol esterification capacity (Figure 2). These IFITM3^+^ microglia exhibited elevated expression of *Acat1* and accumulated cholesterol crystals and lipid droplets, manifesting a distinct pathogenic phenotype characterized by cholesterol metabolic reprogramming. Notably, IFITM3^+^ microglia specifically co‐localized cholesterol crystals and PLIN2^+^ lipid droplets, indicating that this population is overwhelmed by lipid overload and represents a pathogenic state.

Mechanistically, we provide evidence for a link between cholesterol accumulation in IFITM3^+^ microglia and innate immune activation. We suggest that the accumulation of cholesterol within IFITM3^+^ microglia creates a pathogenic state characterized by metabolic stress and robust activation of the STING signaling cascade, driving sustained neuroinflammatory responses [43, 44, 45]. Consistent with this, time‐course analyses showed concurrent increases in cholesterol accumulation, IFITM3 expression, and STING activation in microglia at 3, 7, and 28 days post‐dMCAO (Figures 1A,B, 2G,H, 5C,D). These findings suggest that persistent STING activation and NLRP3 inflammasome signaling are closely associated with the IFITM3^+^ cholesterol‐laden microglial state after stroke (Figures 2 and 3). Additional immunofluorescence analysis revealed that HβCD treatment reduced mitochondrial DNA leakage in microglia, as indicated by decreased TOM20^+^ dsDNA^+^ signal (Figure 5B), supporting a model in which mitochondrial stress and mtDNA release may act as intermediate pathways linking cholesterol accumulation to STING activation. Thus, IFITM3^+^ microglia laden with cholesterol crystals represent a dysfunctional activated population where metabolic stress is converted into chronic inflammatory signaling involving STING signaling.

The downstream consequence of this cascade is severe white matter injury. White matter integrity is essential for functional recovery [46, 47, 48, 49], and we observed a strong spatial correlation between cholesterol crystal deposition and demyelination. STING signaling activation creates a pro‐inflammatory microenvironment that promotes oligodendrocyte death and axonal degeneration. Our findings suggest that white matter injury following PIS may be influenced by the inflammatory response related to uncleared cholesterol crystals in microglia.

We utilized HβCD to mobilize cholesterol. HβCD treatment successfully reduced the cholesterol burden. To confirm the mechanism, we modulated the STING pathway. The protective effects of HβCD were mimicked by a STING inhibitor (C‐176). These experiments indicate that HβCD functions by removing cholesterol, which may serve as the upstream initiator for STING activation. Our results suggest that HβCD serves as a vital adjunctive therapy to facilitate cholesterol clearance, resulting in significant preservation of myelinated axons and improved functional outcomes.

While these findings are suggestive, several limitations must be addressed to facilitate future clinical translation. First, HβCD was employed primarily as a proof‐of‐concept pharmacological tool; however, its pleiotropic effects, complex pharmacokinetics, and restricted blood–brain barrier (BBB) permeability pose substantial challenges for direct clinical application, necessitating the future development of optimized brain‐targeted drug delivery strategies to improve clinical applicability. Similarly, although STING inhibitors such as C‐176 serve as effective laboratory research tools, their in vivo brain penetration, target specificity and rational dosing regimens still require further systematic optimization before clinical use. Second, while AAV9‐mediated STING knockdown yielded powerful neuroprotective effects, the utilization of the F4/80 promoter lacks absolute cell‐type specificity, as F4/80 is shared by both resident microglia and infiltrating monocyte‐derived macrophages, which may weaken the accuracy of cell‐specific intervention. Because our AAV construct does not carry a fluorescent reporter, we are unable to directly visualize or quantitatively assess the cell types that were transduced. Consequently, we cannot fully exclude the possibility of AAV transduction in other myeloid populations, which remains a limitation of the current study. More refined genetic models (e.g., P2ry12‐ or Tmem119‐driven Cre lines) are warranted to definitively dissect the cell‐autonomous role of microglial STING signaling. Third, although we identified IFITM3^+^ microglia as the core pathological population driving abnormal cholesterol deposition and inflammatory activation, the precise upstream molecular signals triggering Ifitm3 expression and the detailed transcriptional regulatory mechanisms controlling cholesterol esterification exclusively within this microglial subset remain incompletely clarified and require further in‐depth exploration. Fourth, formal sample size power calculations were not conducted prior to behavioral functional assessments, which should be fully taken into consideration when interpreting and generalizing the obtained functional recovery outcomes. Fifth, for the scRNA‐seq experiment, tissue from only one animal per group was used. This limits biological replication. Sixth, due to inherent logistical difficulties and limited access to well‐staged clinical peri‐infarct samples, we failed to validate our core conclusions using human stroke tissues and clinical datasets. Combined with the prominent physiological disparities in endogenous lipid metabolism patterns and microglial functional characteristics between rodents and humans, great caution must be exercised when extrapolating rodent experimental results to clinical patient scenarios.

Collectively, our work provides evidence for a distinct pathogenic mechanism involving IFITM3^+^ microglia with aberrant cholesterol esterification after stroke, leading to cholesterol crystal accumulation and STING‐dependent neuroinflammation. However, our findings based on pharmacological and genetic manipulations indicate a probable causal link between cholesterol metabolism and STING signaling. More rigorous genetic modification and targeted ablation assays are needed to solidify this causal mechanism and facilitate subsequent translational research. These findings suggest a potential therapeutic strategy targeting IFITM3^+^ microglia and their cholesterol crystal burden to attenuate neuroinflammation and neurological deficits following cerebral ischemic stroke, and further mechanistic studies are needed to establish causality.

## Author Contributions

Y.C., YX.Z., YH.C., C.C., and T.S. performed the experiments. Y.C., YH.C., T.S., Y.L., and J.Q. analyzed the data. P.L. and YM.Z. wrote the manuscript. C.C., Q.F. YM.Z. and P.L. revised the manuscript. YM.Z. and P.L. designed and supervised the study.

## Funding

This work was supported by National Natural Science Foundation of China, 82525023, W2411087, U22A20295; Fundamental Research Funds for the Central Universities, No. 25X010202804; Shanghai Engineering Research Center of Peri‐operative Organ Support and Function Preservation, No. 20DZ2254200; China Postdoctoral Science Foundation, 2025M782150; Postdoctoral Fellowship Program of CPSF, GZC20261294.

## Ethics Statement

All experiments were approved by the Institutional Animal Care and Use Committee of Cyagen Biosciences (Suzhou) Inc. (approval no. TACU23‐SY214).

## Consent

The author(s) declared that the results/data/figures in this manuscript have not been published elsewhere, nor are they under consideration by another publisher.

## Conflicts of Interest

Peiying Li serves as an Academic Editor of CNS Neuroscience & Therapeutics and is a co‐author of this article. Peiying Li was excluded from all editorial decision‐making processes related to the evaluation, review, and acceptance of this manuscript. Editorial handling and final decisions were conducted independently by another editor to minimize potential bias. The authors declare no conflicts of interest.

## Supporting information


**Figure S1:** UMAP visualization and marker gene expression across annotated brain cell populations.
**Figure S2:** HβCD treatment reduces cholesterol accumulation in microglia after stroke.
**Figure S3:** STING expression in monocyte‐derived macrophages after ischemic stroke.
**Figure S4:** HβCD treatment attenuates ischemic brain injury after stroke.

## Data Availability

The data that support the findings of this study are available from the corresponding author upon reasonable request.scRNA‐seq data from sham‐operated mice (GSM6443690) were generated by our team. Data for ischemic stroke are available under GEO SuperSeries GSE313515.
