# Supplementary material for: Cholesterol Drives IFITM3 + Microglia Activation and Induces STING Mediated Neuroinflammation After Ischemic Stroke
Source: CNS Neurosci Ther. 2026 Aug 11;32(8):e71073. doi: 10.1002/cns.71073 (PMC13459068; doi:10.1002/cns.71073)
Supplement: Supplementary file 1 [file CNS-32-e71073-s001.docx]

**Supplementary Figure S1. UMAP visualization and marker gene expression across annotated brain cell populations.**

**Supplementary Figure S1. UMAP visualization and marker gene expression across annotated brain cell populations.**

(**A**) Feature plots showing the expression of microglial marker genes, including *Cx3cr1, P2ry12*, and *Hexb*. (**B**) Violin plots displaying the expression of canonical marker genes across annotated cell types: astrocytes (*Aqp4*), endothelial cells (*Pecam1*), fibroblasts (*Col1a1*), lymphocytes (*Cd3e*), macrophages (*Ms4a7*), microglia (*Cx3cr1*), monocytes (*Ccr2*), neurons (*Tubb3*), neutrophils (*S100a8*), NK cells (*Nkg7*), and oligodendrocytes (*Mag*).

**Supplementary Figure S2. HβCD treatment reduces cholesterol accumulation in microglia after stroke.**

**Supplementary Figure S2. HβCD treatment reduces cholesterol accumulation in microglia after stroke.**

(**A-B**) Immunofluorescence analysis of free cholesterol in microglia within the peri-infarct region in PBS and HβCD-treated groups 7 days post-dMCAO. (**A**) Representative images showing Filipin III staining in Iba1^+^ cells. Scale bar = 50 μm. (**B**) Quantification of Filipin III area in Iba1^+^ cells (n=4 per group, unpaired *t*-test).

**Supplementary Figure S3. STING expression in monocyte-derived macrophages after ischemic stroke.**

**Supplementary Figure S3. STING expression in monocyte-derived macrophages after ischemic stroke.**

(**A**) Representative immunofluorescence images showing tdTomato, STING and DAPI staining in the peri-infarct region at 7 days after dMCAO. Scale bar = 50 μm. (**B**) Quantification of the percentage of STING signal overlapping with tdTomato⁺ or tdTomato⁻ regions (n=5 per group).

**Supplementary Figure S4. HβCD treatment attenuates ischemic brain injury after stroke**

**Supplementary Figure S4. HβCD treatment attenuates ischemic brain injury after stroke**

(**A**) Representative immunofluorescence images for MAP2 at 7 days post-tMCAO. Quantification of infarct volume (n=7 per group, unpaired *t*-test). Scale bar = 1 cm. (**B**) LFB staining for myelin at 7 days post-tMCAO; upper panel shows low-magnification overview, lower panel displays magnified images. Quantification of myelinated area (n=6 per group, unpaired *t*-test). Scale bars = 0.5 cm (overview), 0.2 cm (magnification). (**C**) Representative multi-regional immunofluorescence images of peri-infarct zones co-stained for MBP, SMI32, and DAPI across CTX, STR, and EC region. Upper panel, low-magnification overview; lower panels, corresponding high-magnification fields from each region in PBS and HβCD-treated mice. Scale bars = 200 μm (overview), 50 μm (magnification). (**D**) Quantification of normalized SMI32/MBP intensity ratio across peri-infarct regions (n=5~6 per group, unpaired *t*-test).
